# Co-Application of *Trichoderma harzianum* and the Strigolactone Analog GR24 Enhances Wheat Tolerance to Cadmium Stress: Effects on Growth, Photosynthesis, Antioxidant Defense, and Cd Accumulation

**DOI:** 10.3390/plants15142236

**Published:** 2026-07-22

**Authors:** Xueping Su, Fangzhao Qin, Cheng Huang, Fasih Ullah Haider

**Affiliations:** 1 Hubei Key Laboratory of Biologic Resources Protection and Utilization, Hubei Minzu University, Enshi 445000, China; suxueping0504@mails.ccnu.edu.cn (X.S.); 13972441467@163.com (F.Q.); 2024102@hbmzu.edu.cn (C.H.); 2 Guangdong Provincial Key Laboratory of Applied Botany, South China Botanical Garden, Chinese Academy of Sciences, Guangzhou 510650, China; 3 State Key Laboratory of Black Soils Conservation and Utilization, Northeast Institute of Geography and Agroecology, Chinese Academy of Sciences, Changchun 130102, China

**Keywords:** *Trichoderma harzianum*, GR24, cadmium uptake, wheat, oxidative stress

## Abstract

Cadmium (Cd) contamination threatens wheat productivity by impairing growth, redox homeostasis, and metal partitioning. Beneficial fungi and phytohormones improve plant stress tolerance, but the combined role of *Trichoderma harzianum* and the synthetic strigolactone analog GR24 in wheat Cd tolerance remains unclear. This study evaluated whether co-application of *T. harzianum* and GR24 enhances Cd tolerance by improving growth, photosynthetic activity, and antioxidant defense, and by reducing Cd accumulation. A greenhouse pot experiment was conducted with two Cd levels (Cd_0_ and Cd_1_), two GR24 treatments (H_0_, without GR24; H_1_, 100 mg L^−1^ GR24), and two *T. harzianum* treatments (TH_0_ and TH_1_; 2 g kg^−1^ soil), using five biological replications. Cd stress markedly inhibited wheat growth, photosynthetic performance, and antioxidant defense while increasing oxidative injury and Cd accumulation in plant tissues. Under Cd stress, the combined GR24-*T. harzianum* treatment increased shoot growth and biomass by 53.04–80.43%, reduced EL, MDA, and H_2_O_2_ by 18.70–30.36%, enhanced SOD, POD, and CAT activities by 24.01–44.50%, and lowered Cd concentrations in roots, shoots, and leaves by 41.09–56.19%. These responses strengthen the treatment’s practical relevance. Collectively, these findings indicate that *T. harzianum*-GR24 co-application offers a promising biological approach to improve wheat resilience and reduce Cd accumulation in wheat tissues.

## 1. Introduction

Heavy metal contamination of agricultural soils is a serious constraint to sustainable crop production [1,2]. These pollutants are persistent and non-biodegradable [3]. These pollutants enter farmland through mining and smelting activities, industrial effluents, wastewater irrigation, and atmospheric deposition. Repeated applications of phosphate fertilizers, manures, and other agricultural inputs provide additional sources of contamination [4]. Among toxic metals, cadmium (Cd) raises particular concern. Cd is highly mobile in soil–plant systems and is readily absorbed by plant roots. It is toxic even at low concentrations and has no known biological function in plants [3,5]. This issue is especially critical in cereals, particularly wheat (*Triticum aestivum* L.), which is consumed by 35–40% of the global population [4]. As a result, Cd contamination directly threatens both crop productivity and food safety [6,7,8,9]. Reported Cd concentrations in rice- and wheat-based cropping systems have exceeded recommended limits by approximately 62% and 81%, respectively. These findings highlight the severity of Cd transfer into staple food crops [10]. At the plant level, Cd toxicity suppresses root growth and disrupts nutrient acquisition. It also impairs chlorophyll biosynthesis and gas exchange, promotes excessive reactive oxygen species (ROS) accumulation, and damages membrane integrity [11,12,13]. Collectively, these effects reduce plant biomass and grain quality [5,14]. In wheat subjected to both Cd and water-deficit stress, Cd concentrations in roots, shoots, and grains reached 8.74–16.15, 4.40–7.45, and 0.15–1.31 mg kg^−1^, respectively. These concentrations showed a negative association with plant growth and physiological performance [12,15]. Therefore, minimizing Cd uptake and toxicity in wheat remains an urgent priority for agricultural sustainability and food safety.

Among biological approaches to managing abiotic stress, *Trichoderma harzianum* is particularly promising due to its plant growth-promoting and stress-protective functions. Its inoculation can improve root development, nutrient acquisition, photosynthetic performance, phytohormone balance, and antioxidant defense [16,17,18]. Under stressful conditions, *T. harzianum* has been associated with greater plant biomass and photosynthetic pigment contents, enhanced catalase (CAT), peroxidase (POD), and superoxide dismutase (SOD) activities, and reduced hydrogen peroxide (H_2_O_2_) accumulation and ionic imbalance [16,19]. These protective responses are particularly relevant to Cd toxicity, which disrupts nutrient homeostasis, photosynthesis, and cellular redox balance. Thus, *T. harzianum* may help alleviate Cd-induced injury by maintaining physiological performance and strengthening antioxidant protection; however, its effectiveness in Cd-stressed wheat remains insufficiently characterized.

Along with beneficial fungi, phytohormones shape plant responses to abiotic stress through physiological and molecular signaling networks [20,21]. Strigolactones (SLs) form a recent class of carotenoid-derived phytohormones. First identified in 1966 from cotton (*Gossypium hirsutum* L.) root exudates, they were found to be germination stimulants for Striga. Only later were their broader roles in plant development and stress adaptation recognized [20,21,22,23]. Studies show that both endogenous SL signaling and application of the synthetic SL analogue GR24 enhance stress tolerance. In drought-stressed winter wheat, GR24 increased root and shoot dry biomass, relative water content, and photosynthetic performance. It also enhanced antioxidant enzyme activities and reduced H_2_O_2_ accumulation [22]. In grapevine (*Vitis vinifera* L.), foliar GR24 increased chlorophyll and relative water content while reducing stomatal opening, EL, MDA, and H_2_O_2_ during drought [23]. Under heavy metal stress, GR24 improved growth, photosynthetic performance, antioxidant activity, and lowered Cd accumulation in switchgrass (*Panicum virgatum* L.) and barley (*Hordeum vulgare* L.) [21,24]. Additionally, rice showed an approximately tenfold increase in endogenous strigolactone levels during mild drought. This response declined under severe, prolonged stress, suggesting that SL-mediated acclimation is dynamic and depends on stress intensity [25]. Strigolactones may improve stress tolerance by regulating abscisic acid-associated stomatal responses and root architecture. They may also influence antioxidant defense, nutrient homeostasis, and interactions with arbuscular mycorrhizal fungi [23].

Despite substantial progress in understanding the individual roles of *T. harzianum* and strigolactones in alleviating abiotic stress, the coordinated effects of these factors on wheat responses to Cd stress remain poorly understood. To our knowledge, the combined application of *T. harzianum* and the synthetic strigolactone analog GR24 to mitigate Cd toxicity in wheat has not been adequately investigated. In particular, it remains unclear whether their co-application can simultaneously improve plant growth, photosynthetic performance, antioxidant defense, and the regulation of Cd accumulation in wheat tissues. Therefore, this study evaluated the individual and combined effects of *T. harzianum* and GR24 on wheat exposed to Cd stress, with particular emphasis on shoot and root growth, photosynthetic traits, oxidative injury, antioxidant enzyme activities, and Cd accumulation. It was hypothesized that the combined treatment would provide greater protection than either treatment alone by improving morphophysiological performance, strengthening antioxidant defense, and reducing Cd accumulation and associated oxidative damage. The findings provide a physiological basis for evaluating this combined biological approach as a potential strategy for improving wheat tolerance to Cd-contaminated soils.

## 2. Materials and Methods

### 2.1. Experimental Site, Soil Preparation, and Cadmium Contamination

The greenhouse pot experiment was conducted at the experimental site of the South China Botanical Garden (SCBG), Guangzhou, China (113.37° E, 23.18° N), from mid-November 2023 to mid-January 2024. The experiment was maintained for 60 days under greenhouse conditions. The study was designed to evaluate the individual and combined effects of *T. harzianum* and the synthetic strigolactone (SL) analog GR24 on wheat growth, photosynthetic performance, oxidative-stress regulation, antioxidant defense, and Cd accumulation under contaminated-soil conditions. The experimental soil was collected from the 0–20 cm surface layer of an agricultural field. After collection, visible plant residues, stones, and coarse material were manually removed. The soil was air-dried at room temperature, gently crushed, passed through a 5 mm sieve, and thoroughly homogenized before use. Before treatment application, a representative soil subsample was analyzed to determine its initial physicochemical properties. The soil had a pH of 5.83 and contained 21.24 g kg^−1^ organic matter. Total nitrogen, phosphorus, and potassium concentrations were 1.38, 0.92, and 17.03 g kg^−1^, respectively, whereas available nitrogen, phosphorus, and potassium were 87.34, 8.96, and 122.43 mg kg^−1^, respectively. The background Cd concentration was 1.98 mg kg^−1^.

Cadmium contamination was induced using cadmium chloride (CdCl_2_) at 30 mg Cd kg^−1^ soil. This Cd level was chosen after a preliminary experiment determined which dose caused physiological stress in wheat without complete seedling mortality. CdCl_2_ was dissolved in distilled water and sprayed onto the soil with mixing to ensure uniform distribution. The Cd-treated soil was incubated for 1 month before sowing to allow Cd to equilibrate and stabilize. During incubation, the soil was mixed regularly, and moisture was kept near field capacity. Non-contaminated soil received the same volume of distilled water, but without Cd. After incubation, each pot was filled with 6 kg of either non-contaminated or Cd-contaminated soil.

### 2.2. Plant Material, Fungal Inoculant, and GR24 Treatment

Seeds of winter wheat (*Triticum aestivum* L. cv. Zhongmai 175) were used as the experimental plant material. Uniform seeds of similar size were selected before sowing. The seeds were surface-sterilized with 1% sodium hypochlorite for 2–3 min, then rinsed several times with sterile distilled water to remove residual sterilizing agent. A commercial formulation of *T. harzianum* marketed under the Hengkun brand was purchased from the Hengkun Agricultural Supplies Official Flagship Store on the Pinduoduo e-commerce platform in China. According to the product label, *T. harzianum* was the declared active microorganism, with a minimum viable count of 1.0 × 10^9^ CFU g^−1^. The product was registered as a microbial fertilizer under Registration No. Microbial Fertilizer (2018) 3447, complied with the Chinese standard GB 20287-2006 [26], and had a labeled shelf life of 12 months. No strain or isolate designation was provided by the manufacturer; therefore, the inoculant is reported at the species level. The carrier composition was also not disclosed on the product label. The formulation was used within its labeled shelf life, although its viability was not independently re-quantified before application. The inoculant was applied once at sowing as a rhizosphere treatment at 2 g kg^−1^ soil. For each pot containing 6 kg of soil, 12 g of the formulation was mixed with a small amount of soil and applied evenly around the seeds. Non-inoculated treatments received sterilized carrier-free soil and distilled water without *T. harzianum*. Synthetic GR24, a strigolactone analog, was obtained from Shanghai Acmec Biochemical Co., Ltd., Shanghai, China. Before conducting the main experiment, a preliminary dose-screening trial was performed to optimize the GR24 concentration for wheat seedlings. Based on the overall improvement in seedling growth and physiological performance, together with the absence of visible phytotoxic symptoms, 100 mg L^−1^ GR24 was selected for the main experiment. Accordingly, the GR24 treatment comprised two levels: H0, without GR24, and H1, with 100 mg L^−1^ GR24. The GR24 solution was applied as a foliar spray at 15 and 30 days after sowing. Control plants received distilled water containing the same concentration of Tween-20 but without GR24.

### 2.3. Experimental Design and Treatment Arrangement

The experiment was arranged as a three-factor factorial trial in a completely randomized design. The three factors were Cd contamination, GR24 application, and *T. harzianum* inoculation. Cadmium was applied at two levels: Cd_0_, non-contaminated soil, and Cd_1_, soil contaminated with 30 mg Cd kg^−1^ soil. GR24 was applied at two levels: H_0_, without GR24, and H_1_, with 100 mg L^−1^ GR24. The fungal inoculation treatment also had two levels: TH_0_, without *T. harzianum*, and TH_1_, with *T. harzianum* at 2 g kg^−1^ soil. The eight treatment combinations were Cd_0_H_0_TH_0_, Cd_0_H_0_TH_1_, Cd_0_H_1_TH_0_, Cd_0_H_1_TH_1_, Cd_1_H_0_TH_0_, Cd_1_H_0_TH_1_, Cd_1_H_1_TH_0_, and Cd_1_H_1_TH_1_. Each treatment was replicated 5 times, yielding 40 experimental units. Wheat seeds were sown directly into pots. After germination, seedlings were thinned to maintain uniform density in each pot. Each pot received the same basal nutrient supply and was irrigated as required to maintain soil moisture near field capacity. Pots were regularly repositioned within the greenhouse to reduce positional effects.

### 2.4. Plant Harvesting and Morphological Measurements

Wheat plants were harvested 60 days after sowing and carefully removed from the pots to minimize root damage. The roots were washed with tap water and subsequently rinsed with distilled water to remove adhering soil particles. For biomass determination, each plant was separated into aboveground shoots and roots. The shoot fraction comprised all aboveground tissues, including stems and leaves. Shoot and root lengths were measured and expressed as cm plant^−1^. Shoot and root fresh biomasses were recorded separately immediately after harvest using an analytical balance. The shoot and root samples were then oven-dried at 65 °C to constant weight, after which their respective dry biomasses were determined. For Cd analysis, the aboveground tissues were further separated into stems (hereafter designated as shoots) and leaves. Thus, Cd concentrations were determined separately in roots, shoots, and leaves.

### 2.5. Determination of Chlorophyll and Carotenoid Contents

Photosynthetic pigments were determined from fresh wheat leaves using the acetone extraction method described by Arnon [27] and Lichtenthaler [28] with minor modifications. Fresh leaf tissue was homogenized in 80% acetone under chilled conditions. The homogenate was centrifuged. The clear supernatant was collected for absorbance measurement. Absorbance was recorded at 663, 645, and 470 nm using a UV–visible spectrophotometer. Chlorophyll *a*, chlorophyll *b*, total chlorophyll, and carotenoid contents were calculated using standard equations and expressed as mg g^−1^ fresh weight. The relative chlorophyll index was measured using a SPAD-502 m (Konica Minolta, Sakai, Osaka, Japan). Measurements were taken from fully expanded wheat leaves. For each plant, SPAD readings were recorded at three points on the leaf blade, avoiding the midrib, and the average value was used for statistical analysis.

### 2.6. Gas Exchange and Chlorophyll Fluorescence Measurements

Gas exchange and chlorophyll fluorescence were measured with a Portable Gas Exchange Fluorescence System GFS-3000 (Walz Heinz GmbH, Eichenring, Effeltrich, Germany). Measurements were taken from fully expanded young leaves under stable greenhouse conditions. Net photosynthetic rate (A), stomatal conductance (gs), transpiration rate (Tr), and intercellular CO_2_ concentration (Ci) were recorded from 09:00 to 11:00 a.m. to minimize variation from diurnal changes. The same leaf position and developmental stage were used across treatments. Chlorophyll fluorescence was measured using the fluorescence module of the same GFS-3000 system. Leaves were dark-adapted for 20–30 min before measurement. The maximum quantum efficiency of photosystem II was expressed as Fv/Fm.

### 2.7. Determination of Oxidative Stress Indicators

Oxidative stress in wheat leaves was evaluated by measuring electrolyte leakage (EL), malondialdehyde (MDA), and hydrogen peroxide (H_2_O_2_). EL was determined following the method of Dionisio-Sese and Tobita [29]. Fresh leaf discs were washed with deionized water and placed in test tubes containing deionized water. After incubation at room temperature, the initial electrical conductivity was recorded. Samples were then boiled to release total electrolytes, cooled to room temperature, and the final electrical conductivity was measured. EL was calculated as the ratio of the initial to the final conductivity. MDA content was determined by the thiobarbituric acid reaction method of Heath and Packer [30]. Fresh leaf tissue was homogenized in trichloroacetic acid and centrifuged. The supernatant was mixed with thiobarbituric acid reagent and heated. After rapid cooling and centrifugation, absorbance was read at 532 and 600 nm with a UV–visible spectrophotometer. MDA content was calculated using the extinction coefficient and recorded as nmol g^−1^ fresh weight. H_2_O_2_ concentration was determined by the method of Velikova et al. [31]. Fresh leaf tissue was homogenized in chilled trichloroacetic acid and centrifuged. The supernatant was mixed with potassium phosphate buffer and potassium iodide. Absorbance was measured at 390 nm with a UV–visible spectrophotometer. H_2_O_2_ concentration was calculated from a standard curve and recorded as µmol g^−1^ fresh weight.

### 2.8. Determination of Antioxidant Enzyme Activities

Antioxidant enzyme activities were determined from fresh wheat leaves. Leaf samples were homogenized in chilled phosphate buffer containing EDTA and polyvinylpyrrolidone. The homogenate was centrifuged at 4 °C, and the supernatant was used for enzyme assays. Superoxide dismutase (SOD) activity was determined according to Giannopolitis and Ries [32] using the inhibition of nitroblue tetrazolium photoreduction. Absorbance was recorded at 560 nm using a UV–visible spectrophotometer. SOD activity was expressed as U g^−1^ FW, with one unit defined as the amount of enzyme required to inhibit nitroblue tetrazolium reduction by 50%. Peroxidase (POD) activity was determined using guaiacol as the substrate, following the method of Maehly [33]. The reaction mixture contained phosphate buffer, guaiacol, hydrogen peroxide, and enzyme extract. The increase in absorbance at 470 nm was recorded using a UV–visible spectrophotometer. Catalase (CAT) activity was determined according to Aebi [34] by monitoring the decline in absorbance at 240 nm due to H_2_O_2_ decomposition. The activities of POD and CAT were normalized to leaf fresh weight and expressed as µmol g^−1^ FW.

### 2.9. Determination of Cd Concentration in Soil, Roots, Shoots, and Leaves

Cd concentration was determined in soil, roots, shoots, and leaves after harvest. Plant samples were washed thoroughly with distilled water, oven-dried at 65 °C to constant weight, and ground into a fine powder. Soil samples were air-dried, gently crushed, and passed through a 2 mm sieve. Dried plant samples were digested with a 3:1 (*v*/*v*) mixture of concentrated HNO_3_ and HClO_4_ using an open-vessel wet-digestion procedure adapted from Zasoski and Burau [35]. Digestion was carried out on a hot plate until a clear solution was obtained. After cooling, the digested samples were filtered and diluted to a known volume with deionized water. For soil Cd determination, air-dried soil samples were digested using the same HNO_3_-HClO_4_ procedure, and the resulting soil Cd concentration was determined by atomic absorption spectrophotometry. Cd concentration in soil and plant digests was determined using an atomic absorption spectrophotometer, Model 3200-C, S/N: KETC0478, Heinz Walz GmbH, Effeltrich, Germany. Cd concentrations were expressed as mg kg^−1^ dry weight for soil, roots, shoots, and leaves.

### 2.10. Statistical Analysis

Data were analyzed using three-way analysis of variance (ANOVA) for a completely randomized factorial design in Statistix 8.1. Cd contamination, GR24 application, and *T. harzianum* inoculation were treated as fixed factors. The model included the main effects of Cd, GR24, and *T. harzianum*, as well as the Cd × GR24, Cd × *T. harzianum*, GR24 × *T. harzianum*, and Cd × GR24 × *T. harzianum* interactions. When significant effects were detected, treatment means were compared using Tukey’s honestly significant difference test at *p* < 0.05. Data are presented as means ± standard errors based on five biological replications. Principal component analysis and Pearson correlation analysis were used to examine relationships among growth, photosynthetic, oxidative-stress, antioxidant, and Cd-accumulation variables.

## 3. Results

**Figure 1:**
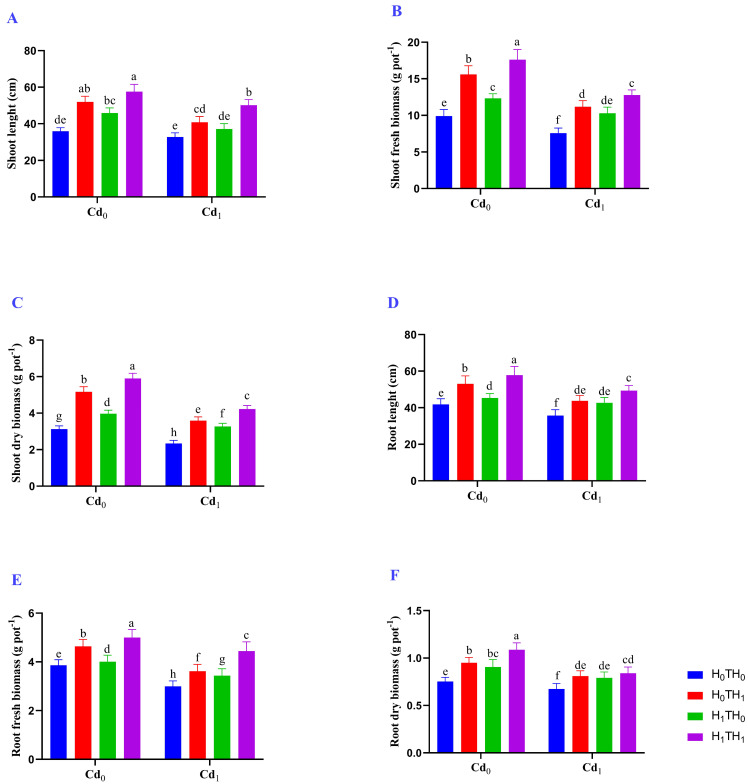
Effects of *Trichoderma harzianum* and GR24 on (**A**) shoot length, (**B**) shoot fresh biomass, (**C**) shoot dry biomass, (**D**) root length, (**E**) root fresh biomass, and (**F**) root dry biomass of wheat grown under non-contaminated and Cd-contaminated conditions. Values are means ± SE (n = 5). Different lowercase letters indicate significant differences among treatment combinations according to Tukey’s HSD test at *p* < 0.05. Cd_0_, non-contaminated soil; Cd_1_, soil contaminated with 30 mg Cd kg^−1^; H_0_, without GR24; H_1_, 100 mg L^−1^ GR24; TH_0_, without *T. harzianum*; TH_1_, 2 g kg^−1^ soil *T. harzianum*.

**Figure 2:**
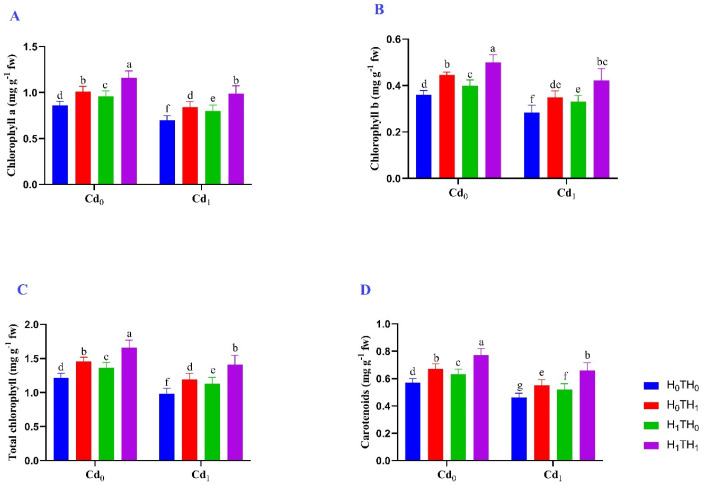
Effects of *Trichoderma harzianum* and GR24 on (**A**) chlorophyll a, (**B**) chlorophyll b, (**C**) total chlorophyll, and (**D**) carotenoid contents in wheat leaves under non-contaminated and Cd-contaminated conditions. Values are means ± SE (n = 5). Different lowercase letters indicate significant differences among treatment combinations according to Tukey’s HSD test at *p* < 0.05. Cd_0_, non-contaminated soil; Cd_1_, soil contaminated with 30 mg Cd kg^−1^; H_0_, without GR24; H_1_, 100 mg L^−1^ GR24; TH_0_, without *T. harzianum*; TH_1_, 2 g kg^−1^ soil *T. harzianum*; FW, fresh weight.

**Figure 3:**
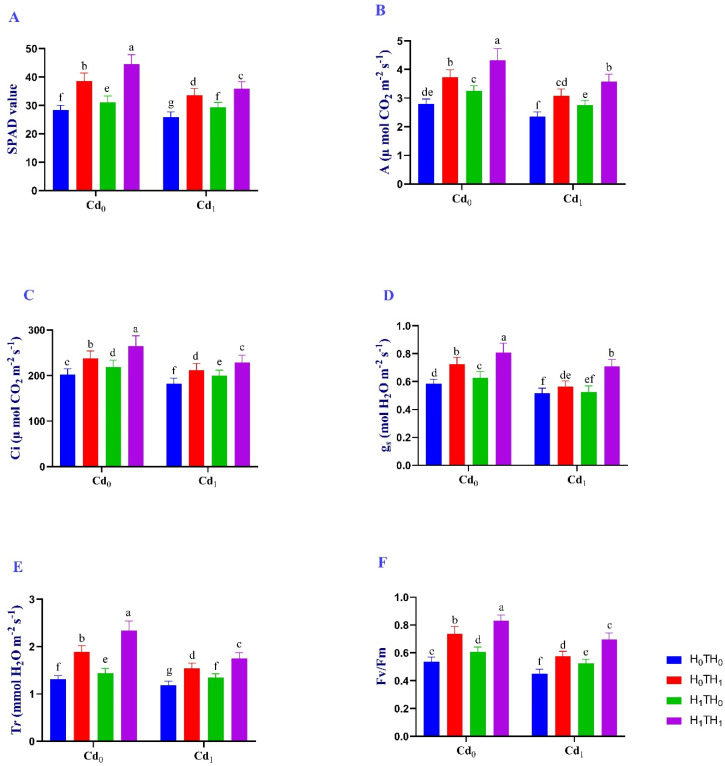
Effects of *Trichoderma harzianum* and GR24 on (**A**) SPAD value, (**B**) net photosynthetic rate (A), (**C**) intercellular CO_2_ concentration (Ci), (**D**) stomatal conductance (gs), (**E**) transpiration rate (Tr), and (**F**) maximum quantum efficiency of photosystem II (Fv/Fm) in wheat under non-contaminated and Cd-contaminated conditions. Values are means ± SE (n = 5). Different lowercase letters indicate significant differences among treatment combinations according to Tukey’s HSD test at *p* < 0.05. Cd_0_, non-contaminated soil; Cd_1_, soil contaminated with 30 mg Cd kg^−1^; H_0_, without GR24; H_1_, 100 mg L^−1^ GR24; TH_0_, without *T. harzianum*; TH_1_, 2 g kg^−1^ soil *T. harzianum*.

**Figure 4:**
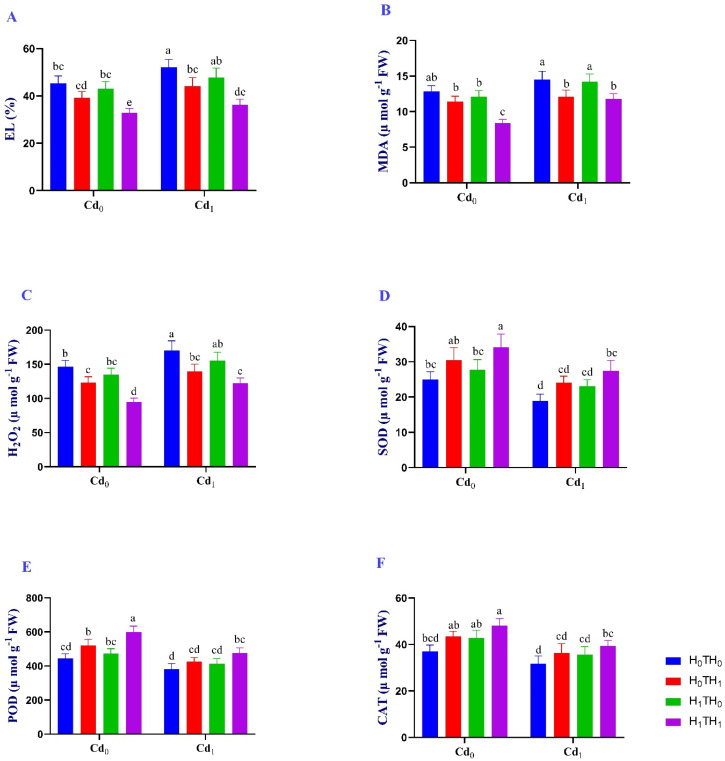
Effects of *Trichoderma harzianum* and GR24 on (**A**) electrolyte leakage (EL), (**B**) malondialdehyde (MDA), (**C**) hydrogen peroxide (H_2_O_2_), (**D**) superoxide dismutase (SOD), (**E**) peroxidase (POD), and (**F**) catalase (CAT) in wheat leaves under non-contaminated and Cd-contaminated conditions. Values are means ± SE (n = 5). Different lowercase letters indicate significant differences among treatment combinations according to Tukey’s HSD test at *p* < 0.05. Cd_0_, non-contaminated soil; Cd_1_, soil contaminated with 30 mg Cd kg^−1^; H_0_, without GR24; H_1_, 100 mg L^−1^ GR24; TH_0_, without *T. harzianum*; TH_1_, 2 g kg^−1^ soil *T. harzianum*; FW, fresh weight.

**Figure 5:**
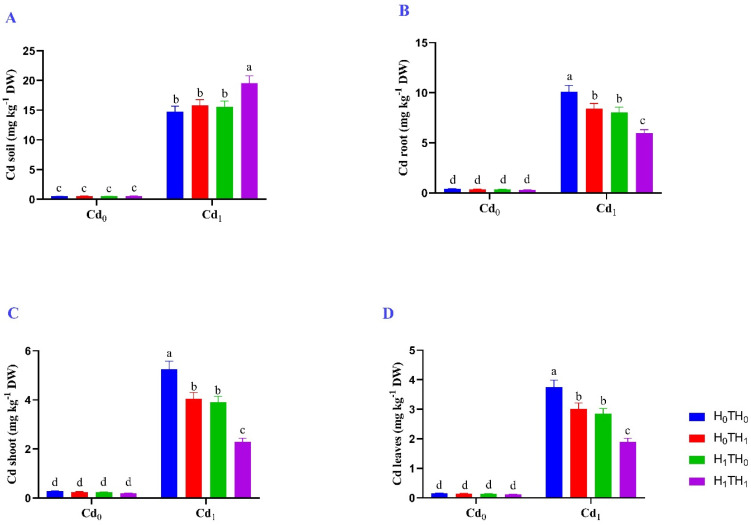
Effects of *Trichoderma harzianum* and GR24 on Cd concentrations in (**A**) soil, (**B**) roots, (**C**) shoots, and (**D**) leaves of wheat grown under non-contaminated and Cd-contaminated conditions. Values are means ± SE (n = 5). Different lowercase letters indicate significant differences among treatment combinations according to Tukey’s HSD test at *p* < 0.05. Cd_0_, non-contaminated soil; Cd_1_, soil contaminated with 30 mg Cd kg^−1^; H_0_, without GR24; H_1_, 100 mg L^−1^ GR24; TH_0_, without *T. harzianum*; TH_1_, 2 g kg^−1^ soil *T. harzianum*; DW, dry weight.

The main effects of Cd, GR24, and *T. harzianum*, together with their two-way and three-way interactions, were evaluated by three-way ANOVA. The *p*-values for the main effects and all two- and three-way interactions are summarized in Supplementary Table S1. Absolute treatment means ± SE are presented in Figure 1, Figure 2, Figure 3, Figure 4 and Figure 5.

### 3.1. Morphological Traits of Plants

Three-way ANOVA showed significant main effects of Cd, GR24, and *T. harzianum* on all six morphological traits. The Cd × GR24 × *T. harzianum* interaction was significant for shoot length (*p* = 0.0236), shoot dry biomass (*p* = 0.0070), and root fresh biomass (*p* = 0.0257), but not for shoot fresh biomass, root length, or root dry biomass (Supplementary Table S1). These interactions indicate that the combined treatment response for the three significant traits depended on the Cd condition (Figure 1). Specifically, Cd stress markedly suppressed wheat growth, reducing SL, SFB, SDB, RL, RFB, and RDB by 8.88%, 23.38%, 25.00%, 14.58%, 22.41%, and 10.52%, respectively, compared with the non-stressed control. The individual and combined application of *T. harzianum* and GR24 significantly improved wheat morphological traits under both Cd-contaminated and non-contaminated conditions. In non-contaminated soil, *T. harzianum*, GR24, and their combination increased SL by 44.40%, 27.40%, and 60.07%, respectively, over the control; SFB by 57.32%, 24.36%, and 77.81%, respectively; and SDB by 65.38%, 27.31%, and 89.10%, respectively. Root traits showed similar trends: RL increased by 26.94%, 8.44%, and 38.42%, RFB by 20.12%, 3.81%, and 29.36%, and RDB by 26.29%, 20.35%, and 44.36%, respectively. Under Cd-contaminated conditions, these treatments promoted growth. Application of *T. harzianum*, GR24, and their combination increased SL by 24.60%, 13.31%, and 53.04%, SFB by 47.23%, 35.46%, and 68.21%, and SDB by 53.42%, 39.74%, and 80.43% compared to the Cd-stressed control. For root traits, RL increased by 22.50%, 19.65%, and 38.15%; RFB by 20.68%, 14.71%, and 48.25%; and RDB by 20.24%, 17.39%, and 24.52%. Collectively, the combined treatment produced the greatest improvement in wheat shoot and root growth under Cd stress.

### 3.2. Chlorophyll and Carotenoid Contents

Three-way ANOVA showed significant main effects of Cd, GR24, and *T. harzianum* on chlorophyll a, chlorophyll b, total chlorophyll, and carotenoids. The GR24 × *T. harzianum* interaction was significant for all four pigment variables (*p* ≤ 0.0316), whereas the Cd × GR24 × *T. harzianum* interaction was not significant for any pigment variable (*p* ≥ 0.3696; Supplementary Table S1). Thus, the pigment response to the combined GR24 and *T. harzianum* treatment did not differ significantly between Cd conditions (Figure 2). Cd stress distinctly decreased chlorophyll a, chlorophyll b, total chlorophyll, and carotenoid contents by 18.60%, 21.11%, 19.34%, and 19.30%, respectively, compared with the non-stressed control. However, the individual and combined application of *T. harzianum* and GR24 substantially increased photosynthetic pigment contents under both Cd-contaminated and non-contaminated conditions. In non-contaminated soil, *T. harzianum*, GR24, and their combination increased chlorophyll a by 17.44%, 11.63%, and 34.88%, respectively, compared with the control. Similarly, chlorophyll b rose by 23.89%, 11.11%, and 38.89%; total chlorophyll by 19.34%, 11.48%, and 36.07%; and carotenoids by 17.54%, 10.53%, and 35.09%, respectively. Under Cd-contaminated conditions, these treatments also had a pronounced beneficial effect. Application of *T. harzianum*, GR24, and their combination enhanced chlorophyll a by 20.00%, 14.29%, and 41.43%, respectively, versus the Cd-stressed control. Similarly, chlorophyll b rose by 23.24%, 16.20%, and 48.59%; total chlorophyll by 20.93%, 14.84%, and 43.50%; and carotenoid content by 19.57%, 13.04%, and 43.49%, respectively. Overall, the combined application of *T. harzianum* and GR24 yielded the greatest increase in photosynthetic pigments, demonstrating that their co-application effectively mitigated Cd-induced inhibition of chlorophyll biosynthesis and sustained the photosynthetic capacity of wheat under Cd stress.

### 3.3. Photosynthesis and Gas Exchange Parameters

Three-way ANOVA showed significant main effects of Cd, GR24, and *T. harzianum* on all photosynthetic and gas-exchange traits. Significant Cd × GR24 × *T. harzianum* interactions were detected for SPAD (*p* < 0.001), intercellular CO_2_ concentration (Ci; *p* = 0.0078), stomatal conductance (gs; *p* < 0.001), and transpiration rate (Tr; *p* < 0.001), but not for net photosynthetic rate (A) or Fv/Fm (Supplementary Table S1). These results indicate that the effects of co-application on SPAD, Ci, gs, and Tr depended on the Cd condition (Figure 3). Cd stress substantially reduced the SPAD value, A, Ci, gs, Tr, and Fv/Fm by 8.69%, 16.05%, 10.30%, 11.25%, 9.35%, and 16.05%, respectively, compared with the non-stressed control. Conversely, individual and combined applications of *T. harzianum* and GR24 markedly enhanced photosynthetic efficiency and gas exchange parameters under both Cd-contaminated and uncontaminated conditions. In uncontaminated soil, *T. harzianum*, GR24, and their combined treatment increased SPAD value by 35.84%, 9.63%, and 57.22%, respectively, compared to the control. Similarly, A increased by 33.15%, 16.12%, and 54.21%, while Ci increased by 17.21%, 7.67%, and 30.72%. Comparable patterns were observed for gs, Tr, and Fv/Fm, which rose by 24.46%, 7.48%, and 38.72%; 44.42%, 9.71%, and 78.85%; and 37.37%, 12.86%, and 54.86%, respectively, in response to *T. harzianum*, GR24, and their combination. Under Cd-contaminated conditions, these treatments also significantly alleviated Cd-induced inhibition of photosynthetic processes. Application of *T. harzianum*, GR24, and their combination increased SPAD value by 29.47%, 13.33%, and 38.67%, respectively, relative to the Cd-stressed control. Similarly, A increased by 30.58%, 17.01%, and 52.16%, while Ci increased by 16.31%, 10.04%, and 25.98%. Moreover, gs increased by 8.95%, 1.74%, and 37.36%, Tr by 29.70%, 13.50%, and 47.46%, and Fv/Fm by 28.01%, 16.30%, and 54.18%, respectively.

### 3.4. Oxidative-Injury Indicators and Antioxidant Enzyme Activities

Three-way ANOVA showed significant main effects of Cd, GR24, and *T. harzianum* on electrolyte leakage, MDA, H_2_O_2_, SOD, POD, and CAT. The Cd × GR24 × *T. harzianum* interaction was not significant for any oxidative-injury or antioxidant variable (*p* ≥ 0.0656). Among the two-way interactions, Cd × GR24 was significant for MDA (*p* = 0.0115), whereas Cd × *T. harzianum* was significant for POD (*p* = 0.0184; Supplementary Table S1). The treatment effects on the remaining variables were therefore primarily associated with the main effects rather than the three-way interaction (Figure 4). Cd stress increased EL, MDA, and H_2_O_2_ by 15.10%, 12.94%, and 16.51%, respectively, relative to the non-stressed control. Under non-contaminated conditions, *T. harzianum*, GR24, and their combination reduced EL by 13.47%, 5.11%, and 27.57%; MDA by 11.25%, 5.81%, and 34.48%; and H_2_O_2_ by 15.60%, 7.94%, and 35.20%, respectively. Under Cd stress, the corresponding reductions were 15.56%, 8.51%, and 30.36% for EL; 16.41%, 2.02%, and 18.70% for MDA; and 18.05%, 8.67%, and 28.18% for H_2_O_2_, respectively.

Cd stress suppressed SOD, POD, and CAT activities by 23.87%, 13.56%, and 14.45%, respectively. In contrast, all applications significantly improved antioxidant enzyme activities. Under non-contaminated conditions, *T. harzianum*, GR24, and the combination increased SOD by 22.14%, 11.19%, and 36.93%, respectively; POD by 17.42%, 6.78%, and 35.37%, respectively; and CAT by 17.24%, 15.46%, and 29.69%, respectively, compared with the control. Under Cd-contaminated conditions, *T. harzianum*, GR24, and their combination increased SOD activity by 26.79%, 21.81%, and 44.50% compared with the Cd-stressed control. POD increased by 10.95%, 7.60%, and 24.01%, and CAT by 14.57%, 12.24%, and 24.30%, respectively. Overall, combining *T. harzianum* with GR24 was most effective at reducing EL, MDA, and H_2_O_2_ levels and enhancing SOD, POD, and CAT activities, indicating improved antioxidant defense and protection against Cd-induced oxidative damage in wheat leaves.

### 3.5. Cadmium Concentrations in Soil and Wheat Tissues

Three-way ANOVA showed significant main effects of Cd, GR24, and *T. harzianum* on Cd concentrations in soil, roots, shoots, and leaves. The Cd × GR24 × *T. harzianum* interaction was significant for soil Cd (*p* < 0.001) and shoot Cd (*p* = 0.0233), but not for root Cd or leaf Cd (Supplementary Table S1). These interactions indicate that the effects of co-application on residual soil Cd and shoot Cd accumulation depended on the Cd condition (Figure 5). Under Cd-contaminated conditions, *T. harzianum*, GR24, and their combined treatment reduced root Cd concentrations by 16.83%, 20.30%, and 41.09%, respectively. Shoot Cd concentrations decreased by 22.86%, 25.71%, and 56.19%, whereas leaf Cd concentrations decreased by 19.63%, 24.00%, and 49.33%, respectively. In contrast, residual soil Cd concentrations were higher by 7.12%, 5.42%, and 32.54% following the application of *T. harzianum*, GR24, and their combination, respectively. This pattern occurred concurrently with lower Cd concentrations in wheat roots, shoots, and leaves and was therefore consistent with altered Cd partitioning between the soil and plant compartments. However, because soil Cd fractions and bioavailable Cd were not measured, these results do not independently demonstrate reduced Cd bioavailability, root uptake, or root-to-shoot translocation.

### 3.6. Principal Component Analysis and Pearson Correlation Analysis

**Figure 6:**
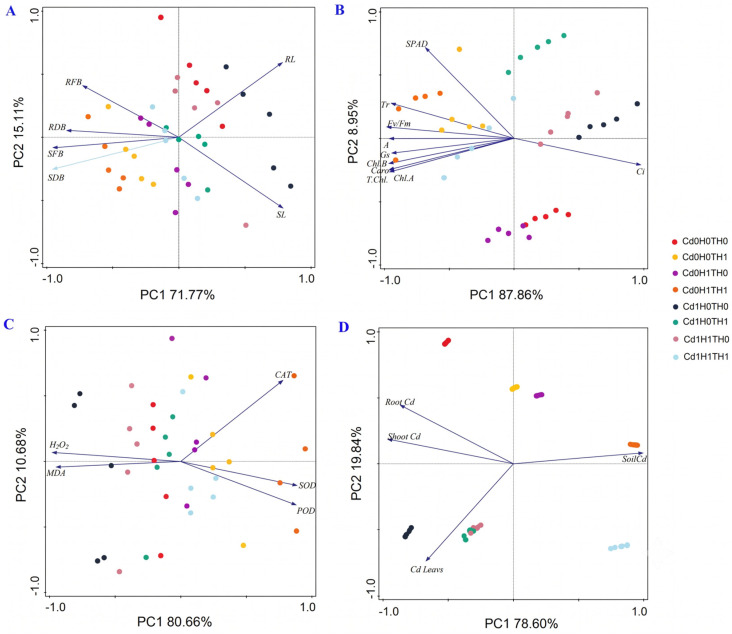
Principal component analysis (PCA) of wheat growth, photosynthetic, antioxidant, and Cd accumulation traits under different treatment combinations. (**A**) Growth traits: shoot length (SL), root length (RL), shoot fresh biomass (SFB), root fresh biomass (RFB), shoot dry biomass (SDB), and root dry biomass (RDB). (**B**) Photosynthetic pigments and gas-exchange traits: chlorophyll a (Chl.a), chlorophyll b (Chl.b), total chlorophyll (T.Chl.), carotenoids (Caro.), relative chlorophyll index (SPAD), net photosynthetic rate (A), intercellular CO_2_ concentration (Ci), stomatal conductance (gs), transpiration rate (Tr), and maximum quantum efficiency of photosystem II (Fv/Fm). (**C**) Oxidative injury and antioxidant traits: electrolyte leakage (EL), malondialdehyde (MDA), hydrogen peroxide (H_2_O_2_), superoxide dismutase (SOD), peroxidase (POD), and catalase (CAT). (**D**) Cd accumulation traits: soil Cd, root Cd, shoot Cd, and leaf Cd. Points represent samples, and arrows indicate trait loading vectors. Cd_0_ = non-contaminated soil; Cd_1_ = Cd-contaminated soil; H_0_ = without GR24; H_1_ = 100 mg L^−1^ GR24; TH_0_ = without *T. harzianum*; TH1 = with *T. harzianum*.

PCA was used to evaluate the integrated responses of wheat growth, photosynthesis, antioxidant defense, and Cd accumulation under different treatments (Figure 6). For growth traits, PC1 and PC2 explained 71.77% and 15.11% of the variance, respectively, with a cumulative contribution of 86.88% (Figure 6A). Treatment groups showed partial separation, indicating distinct growth responses to Cd stress, *T. harzianum* inoculation, and GR24 application. For photosynthetic traits, PC1 and PC2 explained 87.86% and 8.95% of the variance, respectively, for a total of 96.81% (Figure 6B). Clearer clustering was observed among treatments, and the loading vectors of chlorophyll pigments, SPAD, gas-exchange parameters, and Fv/Fm indicated coordinated changes in photosynthetic performance. For oxidative injury and antioxidant traits, PC1 and PC2 explained 80.66% and 10.68% of the variance, respectively, for a total of 91.34% (Figure 6C). Antioxidant enzymes, including SOD, POD, and CAT, were oriented away from MDA and H_2_O_2_, suggesting an inverse relationship between antioxidant activity and oxidative damage. For Cd accumulation traits, PC1 and PC2 explained 78.60% and 19.84% of the variance, respectively, for a total of 98.44% (Figure 6D). Treatment groups formed distinct clusters, indicating strong treatment effects on Cd distribution in soil and plant tissues. Overall, PCA showed that *T. harzianum* inoculation and GR24 application, especially in combination, altered wheat growth, photosynthetic, antioxidant, and Cd-accumulation responses under Cd stress.

**Figure 7:**
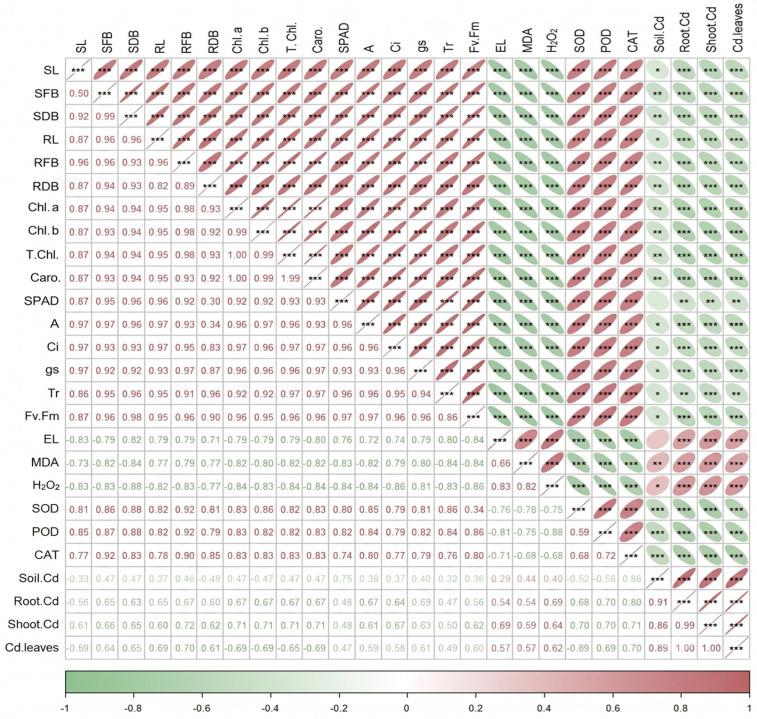
Pearson correlation analysis among wheat growth, photosynthetic, oxidative injury, antioxidant enzyme, and Cd accumulation traits. Growth traits include shoot length (SL), root length (RL), shoot fresh biomass (SFB), root fresh biomass (RFB), shoot dry biomass (SDB), and root dry biomass (RDB). Photosynthetic traits include chlorophyll a (Chl.a), chlorophyll b (Chl.b), total chlorophyll (T.Chl.), carotenoids (Caro.), relative chlorophyll index (SPAD), net photosynthetic rate (A), intercellular CO_2_ concentration (Ci), stomatal conductance (gs), transpiration rate (Tr), and maximum quantum efficiency of photosystem II (Fv/Fm). Oxidative injury and antioxidant traits include electrolyte leakage (EL), malondialdehyde (MDA), hydrogen peroxide (H_2_O_2_), superoxide dismutase (SOD), peroxidase (POD), and catalase (CAT). Cd-related traits include soil Cd, root Cd, shoot Cd, and leaf Cd. Red indicates positive correlations, and green indicates negative correlations. Asterisks indicate significance levels: * *p* < 0.05, ** *p* < 0.01, and *** *p* < 0.001.

Pearson correlation analysis further supported these patterns (Figure 7). Growth traits were positively correlated with chlorophyll pigments, SPAD readings, gas-exchange parameters, and Fv/Fm, indicating that higher biomass production was closely associated with improved photosynthetic performance. In contrast, MDA and H_2_O_2_ were negatively correlated with growth and photosynthetic traits, suggesting that oxidative injury was associated with physiological inhibition. Antioxidant enzymes were positively associated with growth and photosynthetic traits but negatively associated with MDA and H_2_O_2_, suggesting that stronger antioxidant defense contributed to reduced oxidative damage. Cd concentrations in soil, roots, shoots, and leaves were positively correlated with one another but negatively correlated with growth and photosynthetic traits. These relationships indicate that Cd accumulation was associated with growth suppression, photosynthetic impairment, and oxidative stress, whereas *T. harzianum* and strigolactone treatments helped alleviate these effects.

## 4. Discussion

**Figure 8:**
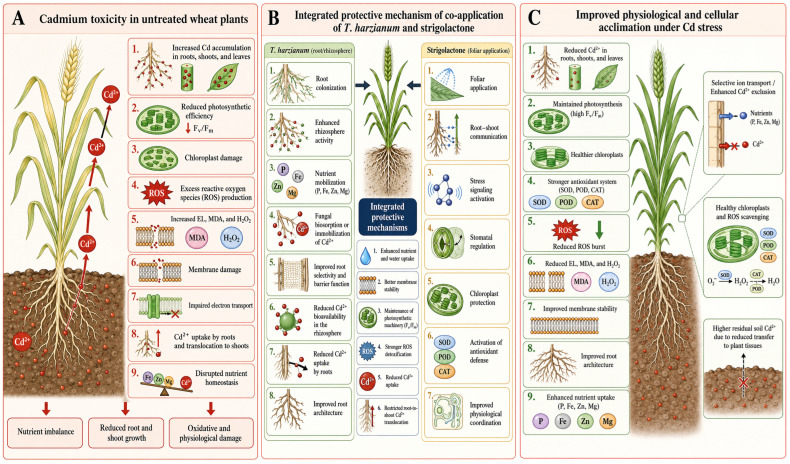
Conceptual model summarizing wheat responses to Cd stress and co-application of *Trichoderma harzianum* and GR24. (**A**) Cd exposure inhibits plant growth and photosynthetic performance and increases oxidative injury and Cd accumulation in wheat tissues. (**B**) Co-application improves growth, photosynthetic performance, and antioxidant enzyme activities while reducing oxidative damage and tissue Cd concentrations. Fungal colonization, rhizosphere modification, nutrient mobilization, fungal Cd binding, altered root selectivity, and regulation of Cd transport are presented as possible contributing mechanisms because they were not directly measured. (**C**) These integrated responses promote improved wheat acclimation to Cd stress. Higher residual total soil Cd occurring together with lower tissue Cd indicates altered soil–plant Cd partitioning but does not confirm Cd immobilization or reduced bioavailability. Note: TH, *Trichoderma harzianum*; GR24, synthetic strigolactone analog; Cd^2+^, cadmium ion; ROS, reactive oxygen species; EL, electrolyte leakage; MDA, malondialdehyde; H_2_O_2_, hydrogen peroxide; SOD, superoxide dismutase; POD, peroxidase; CAT, catalase.

Cd stress strongly disrupted wheat growth, photosynthetic performance, antioxidant homeostasis, and Cd partitioning, whereas the individual and combined applications of *T. harzianum* and GR24 substantially mitigated these adverse effects. The combined H_1_TH_1_ treatment consistently produced the greatest improvement across growth, photosynthetic, oxidative-stress, antioxidant, and Cd-accumulation traits, suggesting that the two treatments produced complementary physiological effects. However, the signaling and molecular processes underlying this combined response were not directly evaluated and therefore remain hypothetical. Cd is a non-essential and highly phytotoxic metal that enters plant roots through transport pathways shared with essential divalent cations, thereby disturbing nutrient homeostasis, membrane integrity, chloroplast function, and redox balance [3,5,36]. Once accumulated in plant tissues, Cd promotes growth inhibition by disrupting root activity, photosynthetic carbon assimilation, and enzymatic ROS-scavenging systems [36,37]. These mechanisms were reflected in the present study by reduced root and shoot growth, lower chlorophyll and carotenoid contents, impaired gas exchange, greater EL, MDA, and H_2_O_2_ accumulation, reduced SOD, POD, and CAT activities, and higher Cd accumulation in wheat tissues under Cd stress (Figure 1, Figure 2, Figure 3, Figure 4 and Figure 5). The proposed schematic model (Figure 8) integrates these responses and suggests that the combined treatment enhanced wheat tolerance primarily by maintaining photosynthetic performance, strengthening antioxidant defense, and reducing Cd accumulation in plant tissues. Improved root-associated processes and altered soil–plant Cd partitioning may also have contributed to this response, although these mechanisms were not directly examined.

The reduction in shoot length, root length, and biomass under Cd stress (Figure 1) indicates that Cd toxicity initially impaired root function and subsequently reduced whole-plant growth. Roots are the primary site of Cd exposure and serve as the main interface controlling Cd entry, nutrient uptake, and water acquisition. Cd can inhibit root meristem activity, disturb cell division and elongation, alter cell wall properties, and reduce root hydraulic and absorptive capacity [36,37,38]. These root-level injuries decrease nutrient and water transport to shoots, ultimately limiting leaf expansion, photosynthesis, and biomass formation. Similar growth suppression has been reported in wheat exposed to Cd, where reduced root and shoot development was associated with impaired physiological activity and higher Cd accumulation [11,12,13,14,15]. Comparable responses have also been reported in barley (*Hordeum vulgare* L.), melon (*Cucumis melo* L.), radish (*Raphanus sativus* L.), mung bean (*Vigna radiata* L.), and Indian mustard (*Brassica juncea* L.), suggesting that root growth inhibition and nutrient imbalance are conserved features of Cd toxicity across plant species [39,40,41,42].

The improvement in wheat growth following *T. harzianum* inoculation suggests that the treatment enhanced root-associated plant performance under Cd stress. Such improvement may be related to fungal effects on root development, nutrient acquisition, and rhizosphere functioning. However, root colonization and persistence of the applied fungus were not verified, and rhizosphere microbial abundance, community composition, and activity were not assessed. Therefore, the contribution of *T. harzianum* to these responses is inferred from the treatment-associated physiological improvements rather than directly confirmed through fungal or microbial analyses. *Trichoderma* species are well-known root-associated fungi that promote plant growth through multiple mechanisms, including root colonization, nutrient mobilization, modulation of phytohormones, induced systemic tolerance, and improved stress-responsive metabolism [43,44,45]. In the present study, improved root length and root biomass under *T. harzianum* application likely increased the absorptive surface available for nutrient and water uptake, thereby supporting greater shoot growth under Cd stress (Figure 1). Evidence from other crops supports this interpretation. *Trichoderma* inoculation improved growth and physiology of mung bean under Cd and Pb toxicity [46], while *T. harzianum*-based treatments improved growth, photosynthesis, nutrient accumulation, and rhizosphere microbial stability in Indian mustard grown in Cd-contaminated soils [47,48,49]. Similarly, *T. harzianum* enhanced stress tolerance in common bean by improving antioxidant defense, nutrient status, and physiological performance [50]. Collectively, these reports suggest that the beneficial effect observed in wheat involved both growth promotion and improved physiological buffering against Cd stress. Root-associated and rhizosphere-mediated processes may have contributed to this response, but their specific involvement requires direct experimental verification.

GR24 application also improved wheat growth under Cd stress, although the response was generally strongest when GR24 was combined with *T. harzianum*. GR24 is a synthetic strigolactone analog used to investigate SL-regulated processes. Endogenous strigolactones are carotenoid-derived phytohormones that regulate shoot branching, root architecture, root-hair formation, leaf senescence, stomatal behavior, and stress adaptation through crosstalk with ABA, auxin, jasmonic acid, and other signaling pathways [20,21,22,23,24,25]. Under Cd stress, this hormone-mediated regulation becomes particularly important because plants must balance growth maintenance with defense activation. In wheat, GR24-mediated SL signaling alleviated Cd toxicity by regulating Cd uptake, nitric oxide signaling, and antioxidant defense-related genes [38]. In barley, GR24 improved Cd tolerance by regulating Cd uptake, antioxidant metabolism, and stress signaling [39], while SL-insensitive barley mutants showed greater sensitivity to Cd and Zn, confirming that SL perception is essential for heavy-metal tolerance [41]. In melon, exogenous GR24 improved root vigor and reduced oxidative injury under Cd stress by reprogramming redox-, jasmonate-, and flavonoid-related pathways [40]. These earlier studies provide a possible mechanistic context for the physiological responses observed in the present experiment. The improved performance following GR24 application was consistent with potential regulation of root development, antioxidant metabolism, and stress signaling; however, hormone crosstalk, signaling molecules, and stress-responsive genes were not measured. The greater growth response under H_1_TH_1_ may therefore reflect complementary physiological effects of the two treatments, but their specific molecular contributions remain to be experimentally determined.

Cadmium stress reduced chlorophyll *a*, chlorophyll *b*, total chlorophyll, carotenoids, and SPAD values (Figure 2 and Figure 3A), indicating damage to both pigment biosynthesis and pigment stability. Chlorophyll reduction under Cd stress may result from Mg (magnesium) and Fe (iron) deficiency, inhibition of chlorophyll biosynthetic enzymes, chloroplast ultrastructural damage, and ROS-mediated pigment degradation [5,36,37]. Carotenoids have an additional photoprotective role because they dissipate excess excitation energy and quench ROS. Therefore, the decline in carotenoids under Cd stress may have increased chloroplast sensitivity to oxidative injury. The recovery of photosynthetic pigments after *T. harzianum* and GR24 application was consistent with improved maintenance of chlorophyll and carotenoid pools under Cd stress. Based on previous studies, *T. harzianum* may have supported pigment retention through root-associated and nutritional effects, whereas GR24 may have influenced antioxidant and stress-response processes [48,49,50,51]. However, chloroplast ultrastructure, pigment-biosynthetic enzymes, and signaling pathways were not examined in the present study; therefore, direct protection of the chloroplast and light-harvesting apparatus cannot be confirmed.

The decline in net photosynthetic rate, stomatal conductance, transpiration rate, intercellular CO_2_ concentration, and Fv/Fm under Cd stress (Figure 3) indicates that Cd imposed both stomatal and non-stomatal limitations on photosynthesis. Lower stomatal conductance and transpiration reflect restricted gas exchange and altered water regulation, whereas reduced Fv/Fm indicates impaired PSII efficiency. Cd can damage photosynthetic processes by disrupting chloroplast membranes, inhibiting electron transport, reducing Rubisco activity, and enhancing ROS formation in chloroplasts [36,37]. Similar photosynthetic inhibition has been reported in Cd-stressed wheat [11,12,13,14,15], barley [39,41], melon [40], radish [42], and mustard [47,48,49]. In the present study, *T. harzianum* and GR24 improved photosynthetic rate, stomatal conductance, transpiration, intercellular CO_2_ concentration, and Fv/Fm, with the strongest response under H_1_TH_1_. This suggests that treated plants maintained more efficient CO_2_ diffusion, water movement, and PSII photochemistry. The improved photosynthetic responses may reflect a combination of better root-associated plant performance and GR24-related regulation of stomatal and photochemical processes, as proposed in previous studies [38,39,40,41]. However, nutrient uptake, plant water status, abscisic acid signaling, ROS-mediated signaling, and individual photochemical components were not directly quantified. These processes should therefore be regarded as possible explanations rather than confirmed mechanisms in the present study.

Oxidative damage was a central component of Cd toxicity in this study. Cd stress increased EL, MDA, and H_2_O_2_ in wheat leaves (Figure 4A–C). Although Cd is not directly redox-active, it indirectly stimulates ROS production by impairing photosynthetic electron transport, mitochondrial metabolism, thiol homeostasis, antioxidant enzyme activity, and membrane stability [36,37]. Increased H_2_O_2_ reflects oxidative pressure, elevated MDA indicates lipid peroxidation, and higher EL reflects membrane permeability damage. These changes, at least in part, explain the reductions in chlorophyll content, Fv/Fm, photosynthetic rate, and biomass observed under Cd stress. The reductions in EL, MDA, and H_2_O_2_ after *T. harzianum* and SL application demonstrate that both treatments lowered oxidative injury. Similar reductions in oxidative damage have been reported after SL treatment in wheat, barley, melon, and radish under Cd or heavy-metal stress [38,39,40,41,42]. Likewise, *Trichoderma*-based treatments reduced oxidative stress and improved stress physiology in mung bean, common bean, and Indian mustard [46,47,48,49,50,51]. These cross-species findings indicate that improved redox homeostasis is commonly associated with microbial- and hormone-mediated heavy-metal tolerance. In the present study, the reductions in EL, MDA, and H_2_O_2_ demonstrate lower oxidative injury, although the upstream signaling processes responsible for these changes were not determined.

The simultaneous increase in antioxidant enzyme activities and decrease in tissue Cd, H_2_O_2_, and MDA should not be viewed as contradictory. Under untreated Cd stress, the lower SOD, POD, and CAT activities suggest that excessive oxidative pressure may have impaired the enzymatic defense system through enzyme inactivation, protein oxidation, nutrient imbalance, or an excessive ROS burden [36,37]. Application of *T. harzianum* and GR24 may have alleviated this inhibition while maintaining or priming antioxidant capacity. SOD converts superoxide radicals into H_2_O_2_, whereas CAT and POD subsequently remove H_2_O_2_ and limit its accumulation to damaging levels. Thus, the higher SOD, POD, and CAT activities under H_1_TH_1_ may have contributed to the observed reductions in H_2_O_2_ and lipid peroxidation, as reflected in lower MDA levels, rather than being solely activated by a greater Cd burden. Previous studies have similarly reported that GR24 and *Trichoderma* treatments improve antioxidant metabolism under abiotic and heavy-metal stress [38,39,40,46,47,48,49,50,51]. Nevertheless, because antioxidant-gene expression and enzyme-protein abundance were not measured, this treatment-induced antioxidant priming remains a plausible physiological interpretation rather than a directly confirmed molecular mechanism.

The pattern of Cd distribution showed that roots accumulated more Cd than shoots and leaves (Figure 5B-D), consistent with their direct exposure to contaminated soil and their role as the first interface for Cd entry into plants. Root retention of Cd can involve binding to cell-wall components, chelation by organic ligands, vacuolar sequestration, and restricted xylem loading [5]. Nevertheless, Cd was also detected in shoots and leaves of untreated Cd-stressed plants, confirming its movement from roots to aboveground tissues. The application of *T. harzianum* and GR24 reduced Cd concentrations in roots, shoots, and leaves, with the greatest reduction observed under H_1_TH_1_. Several processes may explain this response. *T. harzianum* could potentially influence Cd availability in the root zone through fungal surface binding or changes in rhizosphere conditions, whereas GR24 may affect root development and physiological pathways associated with Cd uptake and translocation [38,39,40,41]. Improved membrane stability and nutrient homeostasis may also have contributed to more selective ion uptake. However, Cd bioavailability, fungal Cd binding, rhizosphere chemistry, root selectivity, and Cd-transporter expression were not measured; therefore, these processes remain plausible explanations rather than confirmed mechanisms.

The higher residual soil Cd concentration measured under the amended treatments, particularly H_1_TH_1_, coincided with lower Cd concentrations in wheat roots, shoots, and leaves (Figure 5). This inverse pattern is most appropriately interpreted as a treatment-associated alteration in Cd partitioning between the soil and plant compartments. Because extractable or chemically fractionated soil Cd was not determined, no firm conclusion can be drawn regarding Cd bioavailability or immobilization. Reduced plant uptake, altered root-to-shoot translocation, fungal Cd binding, changes in rhizosphere chemistry, or redistribution among soil Cd fractions represent possible explanations [38,39,40,41,42,49,50,51]. However, these processes were not directly measured. Therefore, the present evidence supports altered soil–plant Cd partitioning rather than confirmed reductions in Cd bioavailability or absolute Cd transfer.

The findings from other plants strengthen the proposed mechanism. In barley, GR24 reduced Cd uptake and improved antioxidant metabolism, while impaired SL perception increased sensitivity to Cd and Zn stress [39,41]. In melon, GR24 reduced Cd-induced oxidative injury and reprogrammed root metabolism [40]. In radish, GR24 combined with biochar improved growth and reduced Cd toxicity [42]. In mung bean, *Trichoderma* inoculation alleviated Cd and Pb toxicity and improved physiological performance [46]. In mustard, combinations of *T. harzianum* with biochar or polyaspartic acid improved root activity, photosynthetic efficiency, microbial network stability, and Cd stress tolerance [47,48,49]. In common bean and Indian mustard, *T. harzianum* improved stress tolerance by regulating antioxidant enzymes, photosynthetic pigments, and nutrient acquisition [50,51]. Although these studies involved different crops and experimental conditions, they collectively indicate that beneficial fungi and GR24 can improve heavy-metal tolerance by interacting to affect plant growth, photosynthetic performance, antioxidant defense, and metal accumulation. Root-associated, rhizosphere, and metal-partitioning processes may contribute to these responses, but their relative importance is likely to vary among plant–soil systems. The present wheat study extends this concept by showing that combining *T. harzianum* with GR24 produces a broader and stronger protective response than either treatment alone.

The conceptual model proposed in Figure 8 integrates the responses observed across Figure 1, Figure 2, Figure 3, Figure 4 and Figure 5. Cd-stressed wheat exhibited restricted growth, lower pigment contents, impaired gas exchange and PSII efficiency, increased oxidative injury, weakened antioxidant defense, and greater Cd accumulation in plant tissues. In contrast, H_1_TH_1_ improved root and shoot growth, maintained photosynthetic performance, enhanced SOD, POD, and CAT activities, reduced oxidative damage, and lowered tissue Cd accumulation. These measured responses may have been supported by complementary root-associated and hormone-regulated processes. *T. harzianum* could potentially influence root development, nutrient acquisition, and rhizosphere conditions, whereas GR24 may contribute to stomatal regulation, antioxidant metabolism, and pathways associated with Cd uptake and transport. However, because fungal colonization, nutrient uptake, rhizosphere properties, root selectivity, and transporter expression were not determined, Figure 8 presents these processes as proposed mechanisms rather than directly demonstrated pathways.

From an agronomic and food-safety perspective, the co-application of *T. harzianum* and GR24 represents a potentially useful biological strategy for wheat cultivation in Cd-contaminated soils. Previous studies cited in this manuscript indicate that the separate application of *T. harzianum* or GR24 can improve plant growth, photosynthetic performance, and physiological tolerance under adverse environmental conditions [16,17,18,19,20,21,22,23,24,38,39,40,41]. However, direct evidence concerning their combined effects on wheat grain yield and grain-quality characteristics under Cd stress remains limited. Because the present experiment ended 60 days after sowing, reproductive development, yield components, final grain yield, grain Cd concentration, and compositional grain quality were not evaluated. Therefore, the observed improvements in vegetative growth and reductions in tissue Cd demonstrate agronomic potential but do not yet confirm successful practical application for grain production or food quality. Full-season field trials should determine whether the combined treatment can maintain grain yield while reducing grain Cd and preserving important quality traits, including grain protein, starch, mineral composition, and thousand-grain weight.

Several limitations should be acknowledged. First, the study was conducted under greenhouse pot conditions, where root volume, microbial dynamics, and Cd behavior may differ from those under field conditions. Second, only one winter wheat genotype, Zhongmai 175, was evaluated; therefore, genotype × treatment interactions could not be assessed, and the findings require validation across genetically diverse cultivars. Third, root colonization and persistence of the applied *T. harzianum* were not verified, and rhizosphere microbial abundance, community composition, and functional activity were not characterized. In addition, only soil Cd concentration was determined; bioavailable Cd, soil Cd fractions, nutrient uptake, root exudates, and Cd-transporter gene expression were not measured. Future studies should integrate fungal-colonization assessment, rhizosphere microbial and biochemical analyses, Cd fractionation, nutrient measurements, root-exudate characterization, and transporter-gene expression. Such analyses, together with multi-genotype and full-season field validation, will clarify the mechanisms and practical value of the combined treatment. Overall, the findings support the hypothesis that fungal inoculation and GR24 application can be integrated to improve wheat resilience under Cd stress.

## 5. Conclusions

This study demonstrates that the co-application of *T. harzianum* and GR24 provided greater protection against Cd stress than either treatment applied individually. The combined treatment improved wheat growth and photosynthetic performance, strengthened antioxidant defense, reduced oxidative injury, and lowered Cd accumulation in roots, shoots, and leaves. These integrated responses identify the co-application of *T. harzianum* and GR24 as a promising biological strategy to improve wheat tolerance in Cd-contaminated soils. Nevertheless, field trials across different wheat genotypes and growing conditions, together with measurements of grain yield and grain Cd concentration, are required before its practical application can be recommended.

## Data Availability

The original contributions presented in this study are included in the article/Supplementary Material. Further inquiries can be directed to the corresponding author.

## Supplementary Materials

The following supporting information can be downloaded at: https://www.mdpi.com/article/10.3390/plants15142236/s1.
