# Supplementary material for: Co-Application of Trichoderma harzianum and the Strigolactone Analog GR24 Enhances Wheat Tolerance to Cadmium Stress: Effects on Growth, Photosynthesis, Antioxidant Defense, and Cd Accumulation
Source: Plants (Basel). 2026 Jul 22;15(14):2236. doi: 10.3390/plants15142236 (PMC13417187; doi:10.3390/plants15142236)
Supplement: Supplementary file 1 [file plants-15-02236-s001.zip › plants-4401657-supplementary.pdf]

Supplementary Table S1. P-values from the three-way factorial ANOVA for the effects of Cd, GR24, *T. harzianum*, and their interactions on wheat growth, photosynthetic, oxidative, antioxidant, and Cd-accumulation variables.

**Variables**	**Cd**	**GR24**	***T. harzianum***	**Cd × GR24**	**Cd × *T. harzianum***	**GR24 × *T. harzianum***	**Cd × GR24 × *T. harzianum***
Shoot length	**<0.001**	**<0.001**	**<0.001**	0.6417	0.0942	0.8480	0.0236
Shoot fresh biomass	**<0.001**	**<0.001**	**<0.001**	0.8143	**<0.001**	0.0366	0.2979
Shoot dry biomass	**<0.001**	**<0.001**	**<0.001**	0.6423	**<0.001**	**<0.001**	0.0070
Root length	**<0.001**	**<0.001**	**<0.001**	0.0241	**<0.001**	0.9304	0.1435
Root fresh biomass	**<0.001**	**<0.001**	**<0.001**	**<0.001**	0.0731	**<0.001**	0.0257
Root dry biomass	**<0.001**	**<0.001**	**<0.001**	0.0027	**<0.001**	0.0219	0.1129
Chlorophyll a	**<0.001**	**<0.001**	**<0.001**	1.0000	0.2275	**<0.001**	1.0000
Chlorophyll b	**<0.001**	**<0.001**	**<0.001**	0.1853	0.1243	0.0316	0.5027
Total chlorophyll	**<0.001**	**<0.001**	**<0.001**	0.4374	0.1263	**<0.001**	0.6966
Carotenoids	**<0.001**	**<0.001**	**<0.001**	0.3696	0.3696	**<0.001**	0.3696
SPAD value	**<0.001**	**<0.001**	**<0.001**	0.0036	**<0.001**	0.0218	**<0.001**
Net photosynthetic rate (A)	**<0.001**	**<0.001**	**<0.001**	0.4750	0.0218	0.1947	0.8700
Intercellular CO_2 concentration (Ci)	**<0.001**	**<0.001**	**<0.001**	0.1156	**<0.001**	0.0163	0.0078
Stomatal conductance (gs)	**<0.001**	**<0.001**	**<0.001**	0.2152	**<0.001**	**<0.001**	**<0.001**
Transpiration rate (Tr)	**<0.001**	**<0.001**	**<0.001**	**<0.001**	**<0.001**	**<0.001**	**<0.001**
Maximum quantum efficiency of PSII (Fv/Fm)	**<0.001**	**<0.001**	**<0.001**	0.2056	**<0.001**	0.0038	0.3800
Electrolyte leakage (EL)	**<0.001**	**<0.001**	**<0.001**	0.3842	0.4143	0.0695	0.8405
Malondialdehyde (MDA)	**<0.001**	**<0.001**	**<0.001**	0.0115	0.7793	0.0574	0.0656
Hydrogen peroxide (H_2O_2)	**<0.001**	**<0.001**	**<0.001**	0.5274	0.9185	0.1395	0.2663
Superoxide dismutase (SOD)	**<0.001**	**<0.001**	**<0.001**	0.7730	0.4751	0.9731	0.6373
Peroxidase (POD)	**<0.001**	**<0.001**	**<0.001**	0.4475	0.0184	0.0860	0.4760
Catalase (CAT)	**<0.001**	**<0.001**	**<0.001**	0.3968	0.4185	0.6294	0.9360

Soil Cd	**<0.001**	**<0.001**	**<0.001**	**<0.001**	**<0.001**	**<0.001**	**<0.001**
Root Cd	**<0.001**	**<0.001**	**<0.001**	**<0.001**	**<0.001**	0.2566	0.2451
Shoot Cd	**<0.001**	**<0.001**	**<0.001**	**<0.001**	**<0.001**	0.0233	0.0233
Leaf Cd	**<0.001**	**<0.001**	**<0.001**	**<0.001**	**<0.001**	0.0916	0.1066

Note: Bold values indicate $p < 0.001$. A = net photosynthetic rate; CAT = catalase; Cd = cadmium; Ci = intercellular CO_2 concentration; EL = electrolyte leakage; Fv/Fm = maximum quantum efficiency of photosystem II; GR24 = synthetic strigolactone analogue; gs = stomatal conductance; H_2O_2 = hydrogen peroxide; MDA = malondialdehyde; POD = peroxidase; SOD = superoxide dismutase; SPAD = relative chlorophyll index; Tr = transpiration rate.
